# Perspective: Opportunities for ultrafast science at SwissFEL

**DOI:** 10.1063/1.4997222

**Published:** 2018-01-08

**Authors:** Rafael Abela, Paul Beaud, Jeroen A. van Bokhoven, Majed Chergui, Thomas Feurer, Johannes Haase, Gerhard Ingold, Steven L. Johnson, Gregor Knopp, Henrik Lemke, Chris J. Milne, Bill Pedrini, Peter Radi, Gebhard Schertler, Jörg Standfuss, Urs Staub, Luc Patthey

**Affiliations:** 1 SwissFEL, Paul-Scherrer Institute, 5232 Villigen PSI, Switzerland; 2 Laboratory for Catalysis and Sustainable Chemistry, Paul-Scherrer Institute, 5232 Villigen PSI, and Department of Chemistry, ETH-Zürich, 8093 Zürich, Switzerland; 3 Laboratoire de Spectroscopie Ultrarapide (LSU) and Lausanne Centre for Ultrafast Science (LACUS), Ecole Polytechnique Fédérale de Lausanne (EPFL), ISIC-FSB, Station 6, 1015 Lausanne, Switzerland; 4 Institute of Applied Physics, University of Bern, Bern, Switzerland; 5 Institute for Quantum Electronics, Eidgenössische Technische Hochschule (ETH) Zürich, 8093 Zurich, Switzerland; 6 Division of Biology and Chemistry, Paul Scherrer Institut, 5232 Villigen PSI, Switzerland; 7 Department of Biology, ETH Zurich, 8093 Zürich, Switzerland; 8 Swiss Light Source, Paul Scherrer Institut, CH-5232 Villigen PSI, Switzerland

## Abstract

We present the main specifications of the newly constructed Swiss Free Electron Laser, SwissFEL, and explore its potential impact on ultrafast science. In light of recent achievements at current X-ray free electron lasers, we discuss the potential territory for new scientific breakthroughs offered by SwissFEL in Chemistry, Biology, and Materials Science, as well as nonlinear X-ray science.

## I. INTRODUCTION

The birth of Femtochemistry, initiated by the late Ahmed Zewail about 30 years ago,^1,2^ made it possible to follow in “real-time” the nuclear motion within molecules, proteins, solids, and liquids. However, these experiments did not resolve the nuclear structure because the probe pulse is in the optical domain. Indeed, optical-domain observables are the intensities and frequencies of electronic transitions, and while delivering valuable information about the temporal evolution of the system, they cannot be translated into structure, except in the case of small molecules (diatomic or triatomic). Therefore, from the early days of ultrafast science, efforts were made to replace the optical probe pulse by pulses of radiation with wavelengths on the order of or shorter than an interatomic bond distance, i.e., X-rays or electrons.^3^ The main challenge in both cases was to generate such pulses.

For electrons, impressive efforts by the group of A. Zewail have opened the field by demonstrating ultrafast scattering, diffraction and microscopy, ultrafast electron energy loss spectroscopy (EELS), and many other methods,^4–16^ while others have improved the time resolution by generating shorter pulses of electrons.^12,17–22^ Ultrafast electron techniques find successful applications in the study of gas phase molecules and materials.^15,16,19,23,24^

In the case of X-rays, ultrashort pulse development has followed two parallel paths. One uses table-top laser sources to generate X-rays. The most widespread implementation of such table top sources for ∼1 Å X-rays uses K_α_ radiation from a rapidly heated solid-state target to generate ∼100 fs pulses at a small set of discrete wavelengths.^25–29^ The other path relies on accelerator-based technologies. Synchrotron sources are attractive for both diffraction and spectroscopy due to their stability and high degree of energy tunability, but they are typically limited to pulse durations of 50–100 ps.^30^ A significant improvement in time resolution (typically 100 fs) was made possible, thanks to the slicing scheme,^31–33^ but at the cost of a dramatic drop in photon flux.

A major development in ultrafast X-ray science has been the advent of X-ray Free Electron Lasers (XFEL)^34^ about ten years ago. This has not only boosted the capability of the existing ultrafast X-ray methods, but has opened the way to new ones that were hitherto impossible. As a consequence, ultrafast X-ray science has witnessed an impressive growth in recent years. Compared to SRs, XFELs have brought about a significant improvement in time resolution (typical pulse widths of <100 fs), along with an impressive increase by several orders of magnitude in photon flux per pulse.^35^ Aside from enabling fs X-ray absorption spectroscopy and X-ray diffraction to be carried out with reasonable data acquisition times,^36^ they have made it possible to perform the first fs-resolved photon-in/photon-out experiments such as X-ray emission (XES)^37,38^ and resonant inelastic X-ray scattering (RIXS) studies on dilute systems^39,40^ and on solids.^41^ It is important to stress that this latter class of experiments can only be carried out at XFELs, making these instruments unique.

The first short-wavelength FEL to be built was FLASH at DESY (Hamburg, Germany) in 2005, which provided vacuum ultraviolet (VUV) to soft X-ray radiation.^42^ The Linac Coherent Light Source (LCLS, SLAC) that came into operation in 2009^43,44^ generates 10^12^ hard X-ray photons per pulse at a repetition rate of 120 Hz, with a pulse duration <100 fs. This development was quickly followed by the XUV FEL FERMI@Elettra (Trieste), which was launched in 2010,^45–50^ and the hard X-ray FEL SACLA in Japan,^51–56^ which was realized in 2011. These machines have proved excellent sources for performing time-resolved X-ray experiments and single-shot structural studies. The FERMI XUV-FEL source shows a dramatic improvement in stability, coherence, and pulse width because of its seedings scheme, and for that reason, it is blazing the trail to the implementation of non-linear X-ray optical techniques, such as the various versions of four-wave mixing studies.^57–59^ In 2017, three more hard X-ray FELs will be commissioned, the European FEL (Hamburg, Germany), SwissFEL (Villigen, Switzerland), and PAL (Pohong, Korea). The field of ultrafast X-ray science has regularly been reviewed in recent years^35,60–68^ covering various aspects of the technical and scientific issues in chemistry, condensed matter physics, biology and non-linear X-ray physics. In this perspective, we will focus on the SwissFEL development and elaborate on the science it will allow in various areas of research.

## II. CONCEPTS, INSTRUMENTS, AND METHODS

**FIG. 1.**
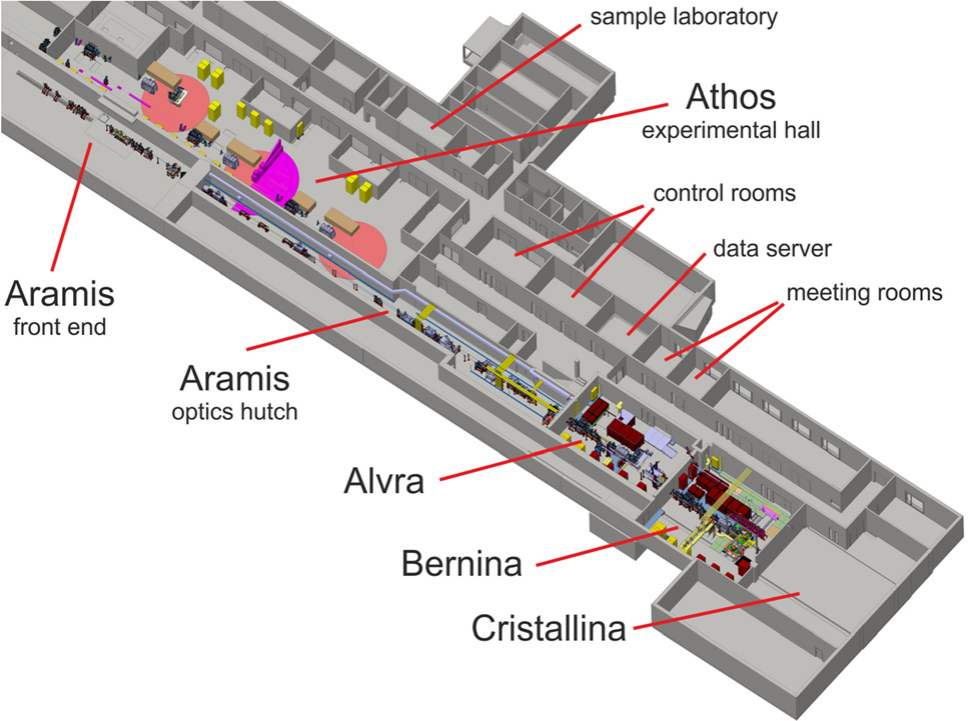
Layout of the experimental area of the SwissFEL building.

The SwissFEL^34^ is located at the Paul Scherrer Institute (Fig. 1), which is a Swiss federal research laboratory and is the home to other national large-scale user facilities. SwissFEL is a compact X-ray free electron laser,^69^ which consists of a linear electron acceleration stage followed by two parallel undulator branches for both hard (1.77–12.4 keV, Aramis) and soft (240–1930 eV, Athos) X-ray operations. It will operate at 100 Hz with both undulator sections simultaneously. SwissFEL will initially operate using Self Amplified Spontaneous Emission (SASE) modes of operation, but plans are drawn to introduce self-seeding operation^70,71^ and several unique operation modes^72–74^ in the future. The design parameters of the facility are listed in Table I. The experimental area includes a large hall for the soft X-ray beamlines and three large hutches for the hard X-ray experiments. The experimental area includes a large hall for the soft X-ray beam lines and three large hutches for the hard X-ray experiments (Fig. 1). Optical lasers will be available for both Athos and Aramis experimental stations.

**TABLE I.** SwissFEL design parameters covering both the soft and hard X-ray branches. Values in brackets are advanced operation modes.

Description	Range
Wavelength (Å)	1 –70
Photon energy (keV)	0.24–12.4
Photons/pulse @ 1 Å	10^11^–10^12^
Pulse energy @ 1 Å (mJ)	1
Pulse duration (FWHM)	1–50 fs (100 as)
Spectral bandwidth	0.05%–0.16% (5%–7%)
Maximum electron energy (GeV)	5.8
Electron bunch charge (pC)	10–200
Repetition rate (Hz)	100

The hard X-ray branch of SwissFEL consists of three beamlines delivering the FEL radiation into separate experimental hutches.^75^ The initial phase of operation will cover the first two experimental hutches, with the third planned for installation in the coming years. The beamlines include several different diagnostics elements to report for every pulse the X-ray spectrum,^76–78^ energy, and beam position.^79,80^ For pump-probe studies, a specialized timing diagnostic tool provides shot-to-shot information on the relative time-delay between the optical laser excitation pulse and the probe X-ray pulse with an accuracy of <10 fs.^55,81^

Each experimental station has access to a highly reliable commercial laser system that provides 20 mJ, 35 fs pulses at 100 Hz and 800 nm.^82^ To allow the excitation wavelength to be tuned from the UV to the IR, both experimental laser systems are equipped with a high-power TOPAS optical parametric amplifier (OPA). Beyond these capabilities, the experimental lasers can also be used to generate THz pulses^83^ or to produce even short pulses (<10 fs) using a hollow-fibre compressor.^84^

The SwissFEL will operate initially with the two Aramis experimental stations. The Experimental station Alvra (ESA) is focussed on ultrafast photochemistry and photobiology, while the Experimental station Bernina (ESB) on condensed matter physics. The X-ray techniques offered by these instruments were accordingly chosen to best suit these topics. The conceptual design reports for all instruments presented here are available on the SwissFEL website.^85^

Alvra focusses primarily on two techniques: X-ray spectroscopy^61,86^ and Serial Femtosecond Crystallography (SFX).^87,88^ These techniques will be applied at two instruments located in line with the X-ray beam: ESA Prime and ESA Flex. Due to the flexible Kirkpatrick-Baez (KB) mirrors, the X-rays can be focussed at either instrument, with the minimum focus of 1.5 *μ*m achieved at ESA Prime. Both instruments can be used with the optical laser for pump-probe experiments, at an anticipated time resolution better than 50 fs.^81^ ESA Prime is a chamber that can be operated under vacuum, He, or neutral atmosphere and combines a 2 D 16M Jungfrau scattering detector^89–91^ with a dual-crystal von Hamos X-ray emission spectrometer. This allows experiments to be performed using both scattering and emission techniques simultaneously, which has proven to be a powerful combination for molecular^38,92^ and protein^93,94^ samples. The chamber can be used with different types of sample injectors, including several types specifically used for protein delivery.^95–99^ SwissFEL has focussed on the development of injectors designed for viscous samples, allowing for minimal sample consumption, while still proving to be appropriate techniques for both time-resolved experiments^100,101^ and room-temperature crystallography experiments at synchrotrons.^102,103^

ESA Flex is a flexible instrument that allows users to build up the experiment as required. It is mounted on a motorized table, so that user-supplied chambers can be installed for the measurement. ESA Flex also includes a configurable X-ray spectrometer that can be mounted in a variety of positions to measure a range of scattering angles, in both vertical and horizontal geometries. When used with large Bragg angles (>85°) and segmented X-ray crystals,^104^ this spectrometer is capable of 100 meV energy resolution.

The experimental station Bernina^105^ is focussed primarily on solid state physics experiments, building on the expertise acquired^106^ at the FEMTO laser-electron slicing source installed at the Swiss Light Source.^33,107^ The primary tool for this will be a 6-circle hard X-ray Kappa-diffractometer with a 1.5M Jungfrau 2D detector mounted on the detector arm, which is capable of both resonant and non-resonant diffraction experiments. In addition to this instrument, Bernina will have a general-purpose station (GPS) upon which a user can mount or build his or her experimental setup. These instruments are mounted on a rail system transverse to the beam to allow them to be moved in and out of position, while maintaining the same X-ray focus position. Both instruments will have access to the optical laser for pump-probe experiments and a large-area 2D 16M Jungfrau detector,^89–91^ which is mounted on a robot arm from the X-ray hutch ceiling to allow for flexible positioning. A specific focus for these instruments is the ability to use strong THz pump fields,^82,83^ which allow for direct excitation of electronic, spin, and lattice dynamics in the condensed phase.^108^

In addition to the two instruments described earlier, a third instrument will be in use at ESB. It is named ESB-MX, and is designed to perform serial femtosecond X-ray protein crystallography measurements in the photon energy range above 5 keV, with protein crystal samples mounted on a solid support.^109^ ESB-MX will include an experimental chamber, equipped with high-speed translation stages to position the samples to within 1 *μ*m accuracy at 100 Hz. The samples will stay either at room temperature or be maintained at cryogenic temperatures by means of a cold gas jet. As sample environment, the choice between air and helium will be offered. The diffraction images will be acquired with the Jungfrau 16 M detector positioned suitably by the ceiling-mounted robot arm. An additional robot for automated sample exchange will permit high throughput measurements.

While the focus has initially been on the installation and development of the hard X-ray branch of SwissFEL, the extension to the soft X-ray Athos branch is also progressing. A sophisticated undulator approach (CHIC)^72^ ensures a flexible photon source for experiments,^73,110^ and a novel undulator design (APPLE X) allows for easy control over polarization and photon energy.^74^ The definition of the three beamlines and their experimental stations is currently in progress in consultation with the SwissFEL user community. Techniques of particular interest include resonant diffraction,^108,111^ X-ray spectroscopy,^39,40,112–114^ X-ray scattering,^115–117^ and electron/ion techniques.^118–121^

## III. APPLICATIONS IN CHEMISTRY

As already mentioned, prior to the advent of the XFEL, tuneable femtosecond X-ray pulses were only available at 3rd-generation light source with a laser-electron slicing facility.^31–33,122^ This scheme was implemented in the hard X-ray range at the Swiss Light Source (SLS),^33^ and was successfully used to probe by X-ray absorption spectroscopy (XAS), the ultrafast structural dynamics of spin cross-over (SCO) in Fe(II) polypyridine complexes undergoing an ultrafast conversion from low spin to high spin upon photoexcitation,^123^ the solvation shell changes upon a photoinduced transition from a hydrophilic to a hydrophobic solvation,^124^ and the trapping of electrons in the photoexcited transition metal oxides.^125^ In the soft X-ray range, Huse and co-workers implemented fs XAS of liquid solutions at the ALS (Berkeley) and investigated the SCO process in Fe(II)-polypyridine complexes at the Fe L-edges^126^ and the N K-edge.^127^

Soon after the advent of the LCLS, the hard X-ray XAS experiments on SCO complexes were repeated demonstrating a dramatic decrease in the data acquisition time due to a much higher photon flux per pulse. The initial issues with the inherent instability of XFEL pulses in terms of photon energy, pulse intensity, and arrival time have, in the meantime, largely been overcome.^36,70,128–131^ In particular, the timing jitter between the laser and the X-rays is now <10 fs. Notwithstanding these improvements, the main contribution to the overall time resolution in ultrafast X-ray spectroscopic measurements (typically ∼125 fs for liquids and ∼70 fs for solids) at XFELs remains the velocity mismatch of the optical and X-ray beams through the sample.^132^

The ability to probe systems with a resolution of ∼100 fs, matched with the very high X-ray photon fluxes per pulse have led to an upsurge of several new experimental opportunities in ultrafast chemistry:^35,133^ (i) studying highly dilute samples; (ii) performing fs photon-in/photon-out experiments, such as X-ray emission spectroscopy (XES) or resonant inelastic X-ray scattering (RIXS); (iii) performing fs X-ray scattering experiments on dilute system; (iv) implementing novel single-shot detection schemes, such as the High Energy Resolution off-resonant spectroscopy (HEROS) methods; (v) extending the toolbox of non-linear techniques to the X-ray range (see Sec. VI), including four-wave mixing and multidimensional spectroscopies, but this time with elemental selectivity and ultrafast time resolution; (vi) performing wave packet dynamics studies. Some of these new capabilities have been demonstrated, while others are still to be proven.

Following the initial proof-of-principle of femtosecond X-ray Absorption Near Edge Structure (Fs-XANES) on SCO Iron(II) molecular complex at an XFEL,^36^ Lemke *et al.*^134^ observed, for the first time by XAS, coherent wave packet oscillations induced by the impulsive SCO in solution, in line with the previous optical-only ultrafast studies.^135,136^ More recently, Collet and co-workers extended these studies to SCO molecular crystals.^137^ At SACLA, studies were performed on an Fe-complex, [Fe^III^(C_2_O_4_)_3_]^3+^ in solution,^138^ delivering insight into the photoinduced electronic and structural changes in the system, while the photoinduced charge carrier dynamics was investigated in the case of WO_3_ nanoparticles,^139^ and of TiO_2_.^140^ The latter studies repeated those by Santomauro *et al.*^125^ using the slicing scheme, but this time with a greatly enhanced S/N, which allowed them to detect a factor of 3 between the electron trapping time (∼100 fs) and the ensuing structural relaxation of the trap (∼300 fs). Finally, following the fs Fe K-edge XAS experiment on Carboxymyoglobin (MbCO) at single probe energies,^132^ polarized fs Co K-edge XANES on Vitamin B12 was for the first time reported, probing the structural evolution of the protein.^141^ In another study, Mara and co-workers reported an ultrafast energy-resolved XAS and XES study of photoexcited ferrous Cytochrome C.^142^ These various studies illustrate the diversity of systems one can investigate by fs X-ray spectroscopy at XFELs.

As already mentioned, fs photon-in/photon-out experiments can only be carried out at XFELs. Nilsson and co-workers^113,143^ demonstrated the ultrafast laser pump/XES probe in the soft X-ray range looking at the electronic structure of a transiently populated, weakly adsorbed state in CO desorption from Ru(0001). This first study was followed by a fs soft X-ray XAS experiment that investigated the formation of the CO_2_ molecule starting from CO and O on Ru(0001).^112^ In the hard X-ray range, Zhang *et al.* exploited the high sensitivity of XES to spin states^144,145^ and mapped with 150 fs resolution the SCO relaxation cascade in photoexcited [Fe(bpy)_3_]^2+^.^37^ Very recently, Wernet and co-workers^39,40^ investigated the reaction dynamics of the transition-metal complex Fe(CO)_5_ in solution, using fs RIXS in the soft X-ray range, stressing the relative importance of different spin channels in the photochemistry of Fe(CO)_5_.

The aforementioned examples dealt with spectroscopic signals, but the ability to perform fs X-ray scattering (XRS) studies in solution is also one of the unique features of XFELs. Furthermore, because it uses a fixed incident energy, it can be combined with XES in the same experiment, as was first demonstrated at synchrotrons.^146^ Haldrup *et al.*^147^ implemented this approach in a study of [Fe(bpy)_3_]^2+^, this time looking at the solvent response upon the SCO. This type of study was recently extended to different classes of molecular solutes, such a bimetallic ones by van Driel *et al.*, to look at the coordination of the excited solute with a solvent molecule.^148^ It was also exploited by Canton *et al.*^38^ at SACLA to characterize the non-equilibrated electron transfer (ET) dynamics in donor–acceptor molecular assemblies consisting of a light-harvesting, ruthenium (Ru)-based chromophore linked to an optically dark cobalt (Co) electron sink by a bridge that mediates the ultrafast ET.

A simulation by Penfold *et al.*, on a model system consisting of di-iodine in solution,^149^ had predicted the possibility to perform fs XRS of wave packet dynamics by exploiting the polarization properties of the pump and probe pulses. Fs XRS was recently realised by Biasin *et al.*^150^ who probed the structural dynamics upon photoinduced SCO in aqueous [Co^II^(terpy)_2_]. Their analysis showed that photoexcitation leads to impulsive elongation of the Co-N bonds, giving rise to coherent Co-N bond length oscillations. This first demonstration of vibrational wave packets by fs XRS in solution opens the way to more systematic studies on molecular systems, as the tools are being refined and the data treatment becomes more routine.

The aforementioned short review of the literature of the last four years or so shows that the stage is set for new opportunities to come. The ability to perform highly sensitive X-ray spectroscopic or scattering experiments now opens the door to look into subtle effects or phenomena such as solvation, wave packet dynamics, or low yield and/or competing photochemical channels. As invoked earlier, femtosecond photon-in/photon-out experiments can only be carried out in such machines, making them unique to this purpose. For proteins (see also Sec. V), XES is already very much used in the static mode in the so-called Serial Femtosecond Crystallography (SFX),^93,151–153^ and extension to the time-resolved domain is underway. For solid materials (see also Sec. IV), specifically single crystals, RIXS enables the Q-dependent study of collective excitations because a finite momentum can be transferred to the system from the X-ray photon.^154,155^ In a very recent work, Hill and co-workers reported the first fs magnetic RIXS after photo-doping the Mott insulator Sr_2_IrO_4_, and directly determined its magnetization dynamics.^41^ Although concerning solid materials, such studies are also of interest to photovoltaics and photocatalysis when it comes to transition metal oxides^125,156^ and perovskites,^157^ as the bulk properties of these materials need to be nailed down, which means studying single crystals as was recently demonstrated in the case of TiO_2_.^158^

**FIG. 2.**
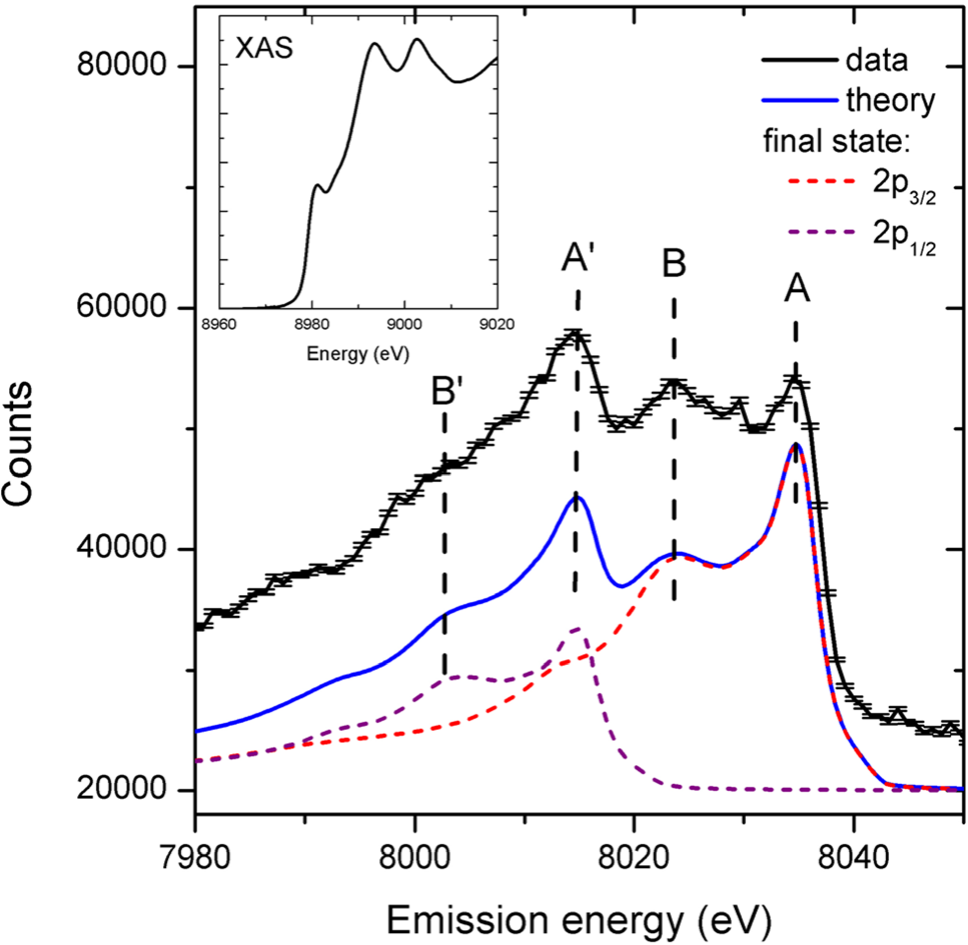
HEROS spectra of Cu metal for 2000 self-seeded shots (black curve). The error bars represent the standard deviation of the total counts. For comparison, we plot the calculated spectrum using the Kramers-Heisenberg relation and a Cu K-edge XAS spectrum recorded at a synchrotron facility shown in inset. The calculated curve represents the sum of two spectra relating to the final electronic states of 2p_3/2_ and 2p_1/2_. Reproduced with the permission from Struct. Dyn. **1**, 021101 (2014). Copyright 2014 AIP Publishing LLC.

A promising approach to record single shot absorption spectra is the so-called High Energy Resolution Off-Resonant Spectroscopy (HEROS) method, which allows measurements of a scattered X-ray spectrum that represents the unoccupied density of states. It is complementary to XES that probes the occupied density of states. The idea of using off-resonant excitation is similar to the pre-resonance Raman spectroscopy in the optical domain. HEROS requires monochromatic photon energies for the incoming beam. One way to generate this type of beam at an XFEL is the so-called “self-seeding” method,^160^ which allows the FEL to produce a narrow energy bandwidth beam with more stable characteristics than during normal SASE operation with a monochromator. Szlachetko, Milne, and co-workers^159^ exploited this option at the LCLS and recorded the first static HEROS spectrum using a single fs hard X-ray pulse (Fig. 2). This elegant alternative to XAS has the advantage that it can be combined with X-ray scattering experiments. The first application of HEROS to study dynamics in an ultrafast pump-probe experiment has yet to be performed.

One of the attractive features of X-ray spectroscopy, and in particular, photon-in/photon-out methods, is their ability to observe optically “dark” states.^37,38,123,161^ A good example is provided by the states that mediate a bypass of the S_1_ state of diplatinum complexes straight into the lowest T_1_ state, upon excitation of high lying electronic states.^162^ Several such examples exist in the photophysics of metal complexes^163,164^ or biosystems.^165^ Another important perspective, once multiple X-ray pulses can be generated, is to interrogate several centres of a photoexcited system, and investigate issues such as two-centre electron transfer, which were reported by ps XAS on Rhenium-based molecular complexes.^166^ In general, transition metal complexes have been one of the main playgrounds for the implementation of time resolved X-ray spectroscopies^35,167^ because of the many fundamental questions they raise, in particular the rich correlated charge, spin, and structural dynamics, and this will continue with XFELs.

Many, if not most, chemical processes are activated by heat rather than light. In these cases an initializing trigger for ultrafast X-ray studies should mimic a thermal excitation. That means a resonant or off-resonant excitation of molecular vibrations on an ultrafast time scale. Since temperature is defined as an equilibrium state that is mainly not reached on ultrafast time scales the excitation mechanism has be adapted to the given experimental application. Ideally the main energy should be pumped into interatomic vibrations along the reaction coordinate. However, dissipation mechanisms and their timescales are mostly unknown and therefore need careful consideration. Moreover, it has to be ensured that no parasitic processes such as electronic excitation and/or charging are present especially when dealing with off-resonant excitation schemes. For instance, the use of low energy radiation, i.e., single cycle THz pulses, has been proposed to resonantly pump energy into the vibrational mode along the reaction coordinate.^168^ Such pulses are on the order of one 1 ps duration and are extremely broad in frequency. Since temperature is defined as an equilibrium state that is mainly not reached on ultrafast time scales the excitation mechanism has to be adapted to the given experimental application. Thus, such pulses are ideal candidates to excite low frequency thermal like vibrations on an ultrafast timescale to overcome the excitation barrier and trigger the chemical reaction. First experiments have shown that CO oxidation on ruthenium can be induced by THz radiation.^169^ Recently, ultrafast X-ray emission and absorption experiments at LCLS showed the time evolution of surface bond breaking^113^ and could probe the transition state region of a surface catalytic reaction triggered by an optical laser pulse.^112^ The planed THz source at the SwissFEL relies on optical rectification in organic crystals such as DAST, DSTMS, OH1, and HMQ-TMS. The SwissFEL laser group has recently pioneered broadband and intense THz generation from such organic crystals.^83,170–172^ The spectral bandwidth can stretch from 0.5 THz to 12 THz, achieving pulse energies between 10 *μ*J and 50 *μ*J. Further approaches towards even higher pulse energies and field strength are under development by pumping large area organic crystals^173^ directly via a Cr:forsterite laser system.^174,175^ For narrowband excitation, a variety of metamaterial filters will be available acting as high-pass, low-pass, or band-pass filter.

## IV. APPLICATIONS IN CONDENSED MATTER PHYSICS

As mentioned in Sec. I, the development of the electron-beam slicing technique made possible by a variety of femtosecond time-resolved experiments at accelerator-based X-ray sources, with many early studies focusing on dynamics in condensed matter systems. An early example of a slicing experiment on solid materials is a soft X-ray spectroscopic study of the metal-insulator transition in the prototypical VO_2_ material.^176^ Later on, the use of slicing sources in condensed matter physics produced a series of results, which were focused predominantly on X-ray diffraction (XRD) and X-ray magnetic circular dichroism (XMCD) in the hard and soft X-ray regimes, respectively. Hard XRD has been used to investigate displacive coherent phonon excitations,^33,177–180^ phonon squeezing,^178^ and to study the structural response of ultrafast electronic phase transitions in several materials.^180–188^ In the soft X-ray regime, extensive work was performed on ultrafast demagnetization of ferromagnets^189,190^ and the study of all-optical switching, for which a transient ferromagnetic state has been observed.^191^

**FIG. 3.**
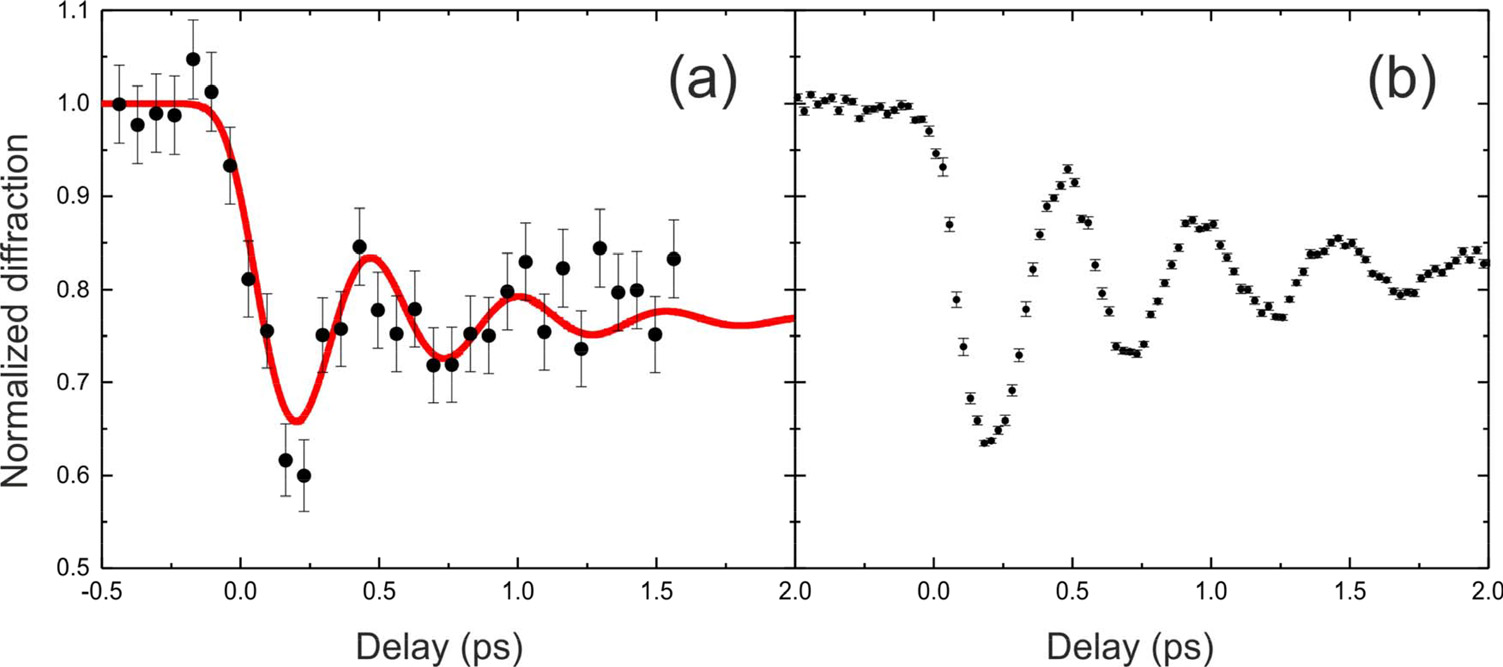
Dynamical response of the (5 –5/2 2) superlattice reflection in the ground state of a thin film of La_1-x_Ca_x_MnO_3_ (x = 0.58) to ultrashort pulse excitation at 800 nm. The observed dynamics for an intermediate fluence of ∼1 mJ/cm^2^ are dominated by a strong ∼2.5 THz oscillation, which is the slowest of several coherent optical modes observed when these materials are excited with very short optical pulses (a) data obtained at the SLS slicing source^192^ [reproduced with permission from Beaud *et al.*, Phys. Rev. Lett. **103**(15), 155702 (2009). Copyright 2009American Physical Society] and; (b) same, re-measured four years later at the XPP instrument at LCLS.

In the meantime, pump-probe experiments at XFELs have been used for many studies using traditional single-crystal diffraction on solids in the hard X-ray regime with different pump schemes. A comparison of the dynamical response of a superlattice reflection in the ground state of a thin film of La_1-x_Ca_x_MnO_3_ (x = 0.58) to ultrashort pulse excitation at 800 nm from the data taken with the slicing source and from XPP at the LCLS is shown in Fig. 3. The figure illustrates the tremendous progress made in hard X-ray source development in a couple of years. The scan measured at the FEMTO slicing source at SLS (a)^192^ lasted over 24 h compared to only 5 min (Ref. 193) required at LCLS to obtain the transient shown in (b) for the very same sample, with much better statistics and time resolution. However, it has even been shown that in certain cases that experiments performed at these low flux sources can compete with experiments at XFELs, even though the XFEL offers significant better time resolution and photon flux.^194,195^

**FIG. 4.**
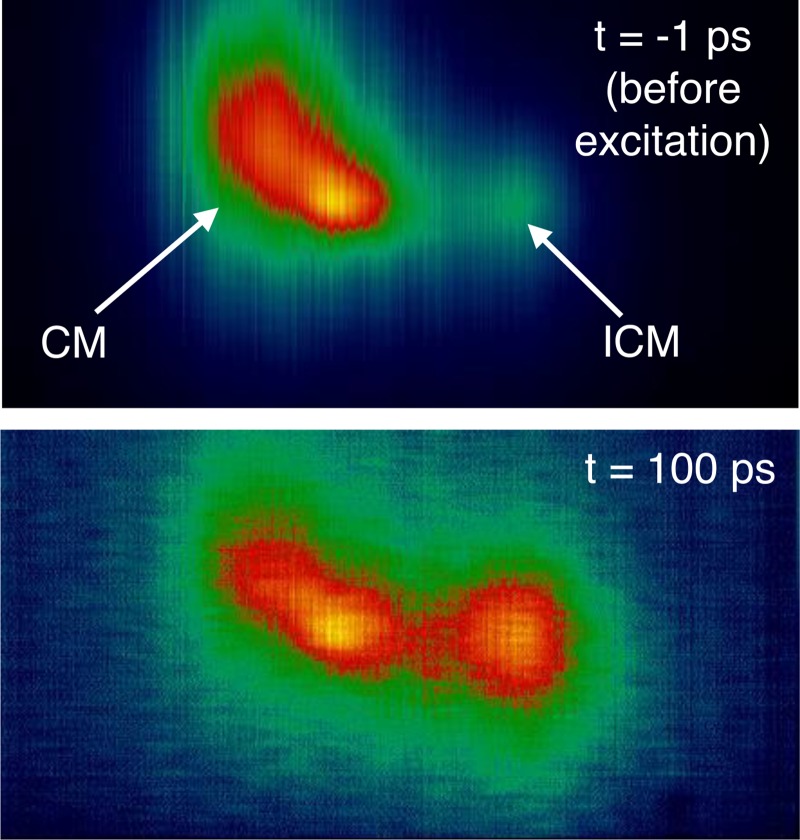
Images of resonant X-ray diffraction on CuO taken from Ref. 111, 1 ps before excitation (upper panel) and 100 ps after excitation (lower panel). Reproduced with permission from Johnson *et al.*, Phys. Rev. Lett. **108**(3), 037203 (2012). Copyright 2012 American Physical Society.

At the same time, new techniques have emerged, which take better advantage of the uniquely high photon flux per pulse available at XFEL facilities. Prominent examples include resonant magnetic diffraction that has been applied to study the suppression of magnetic order in different shells of Ho,^196^ or resonant charge scattering that has been employed to investigate electronic transitions in PCMO.^193^ The latter study showed that the ultrafast charge and orbital order transition can be described with a Landau-like time-dependent order parameter.^193^ In addition, diffuse X-ray scattering has been utilized to determine the phonon dispersion in the time domain^197^ and RIXS has been implemented to investigate the temporal dependence of the dispersion of magnetic excitation exemplified in the hard X-ray regime of an Iridate.^41^ In the soft X-ray regime, a series of experiments in the field of correlated electron systems has been performed using pump-probe resonant diffraction. An example is presented in Fig. 4, showing the relative enhancement of a magnetic satellite reflection of CuO taken at the Cu L_3_ edge after photoexcitation. These data have been used to detect and characterize the ultrafast magnetic phase transition in an antiferromagnet.^111^

Correlated systems studied with soft and hard X-rays at XFELs include cuprates,^188,195,198,199^ charge density wave systems,^200^ manganites,^193,201,202^ nickelates,^203–205^ magnetite,^206,207^ and multiferroics.^108,111,208,209^ Early experiments using XMCD contrast have been limited by the restricted amount of circular light polarization available at the LCLS, but nonetheless important information on the local behaviour of all optical switching in GdCoFe intermetallics has been obtained.^210^ With the installation of a new variable-polarization delta undulator at LCLS, experimenters now have access to a much more flexible source of circular polarization.^211^

The future prospects for ultrafast X-ray experiments on the dynamics of condensed matter physics show considerable promise. This field remains exciting as many of the X-ray techniques have not yet been explored or were limited to proof-of-principle experiments. In addition, the speed of the technical developments will further accelerate in the next years with the new XFEL sources coming online, along with the LCLS upgrade programme, all of which serve to push the available photon flux and implement higher repetition rates. In the following, we concentrate on opportunities of SwissFEL with its pump-probe instrument Bernina, which focuses on the study of solid materials in the hard X-ray regime.

One important avenue to explore here is the ability to excite materials in a controlled way, for example, by directly pumping a particular phonon with tuned infrared pulses, and then to probe the succeeding structural, magnetic, or electronic responses in real time. Of particular interest is to see what happens during the variation of the electric field within the pulse. Pioneering experiments along these lines have already been performed in the mid-IR region on cuprates^198^ and manganites,^202^ although they do not yet have the ability to follow the dynamics driven by the E-field within a cycle of the pump pulse. This has been achieved at lower frequencies using phase-stable few-cycle excitations, which were used to excite polar modes in quantum paraelectric SrTiO_3_^212,213^ as well as to excite coupled phonon-magnon modes (electromagnons) in multiferroics.^108^ The current plans for the SwissFEL envision flexible sources of phase-stable mid-IR pulses covering the most important 5–20 THz range that will enable the manipulation of material properties on ultrafast timescales in a much more controlled way. Recent works on studying the transient states created by such excitations have produced some surprising results, including observations in several materials of what appears to be a light-induced superconducting-like state^214^ and the possible switching of ferroelectric polarizations.^215^ By combining such excitations with ultrashort X-ray pulses we will be able to study qualitatively how the atomic motions driven by light alter the structure and set the stage for these novel transient states.

Another significant challenge ahead of us is to improve the overall time resolution in pump-probe experiments. At present, the current standard is approximately 70 fs at LCLS made possible using a timing tool that measures shot-to-shot the arrival time jitter between the FEL and the pump laser pulses with an accuracy of 5 fs. The scientific drive for improving the overall time resolution in condensed matter is manifold. First, it will be important to access faster coherent motions, either excited by optical pumps or by the direct excitation with a phase stable mid-IR pump as mentioned before. In the first case, both the optical pump and the X-ray pulse length have to become shorter, and new concepts for timing diagnostics need to be developed, which work with weaker X-ray pulses. This increased time resolution is critical for an understanding of many processes in complex oxides where the most important vibrational modes have periods of 50 fs or even less.^193^ With a time resolution below 10 fs it will be possible to separate contributions of nuclear motion from pure electronic dynamics. It is also important to time-resolve the dependence of the individual nuclear motions within the cycle of the driving electric field. For that purpose, the X-ray pulse length has to be short compared to the oscillation period of the pump pulse. Shorter X-ray pulses are available only at the expense of their intensity implying again a timing tool that works at these weak photon fluxes. When improving the time resolution significantly, one will naturally approach the regime of non-linear X-ray science discussed later (see Sec. VI). To have sufficient signals for the study of even faster processes, it will naturally lead to the entanglement of non-linear X-ray science into applications in condensed matter.

Another important development in XFEL pump-probe experiments is to enable more flexible sample environments. In particular, experiments are performed at this stage mainly at 100 K or higher for hard X-rays and above 20 K for soft X-rays, with very few exceptions. However, for strongly correlated electron systems, a larger temperature and magnetic field range is required to address many of the interesting and fundamental questions in this field. In addition, more advanced X-ray techniques need to be implemented to go beyond first proof-of-principle experiments to assess whether they are more generally useful. In particular, fs RIXS has to be further explored, as it has the potential to study the electronic excited states directly.^41^

## V. APPLICATIONS IN STRUCTURAL BIOLOGY

Modern X-ray sources have had a tremendous impact on the biological sciences and particularly on our understanding of biological macromolecules at the atomic level. Precise knowledge of the interactions between proteins and their ligands provided critical insights into the mode of action of numerous pharmaceutical molecules. At the time of writing, the protein data bank has gathered 129 942 entries, the vast majority derived from protein crystallography at 3rd generation synchrotron sources. This success is the culmination of decades of gradual improvements, including the development of fast, low noise detectors and high degrees of automation in sample handling and data analysis. Yet one fundamental obstacle remains: For each macromolecule, there is a dose limit it can take before the X-ray radiation damage alters its structure. Especially at 3rd generation synchrotron sources with their high repetition rates the dose limit is reached within a few seconds. The dose limit reduces the obtainable resolution, but in many cases the radiation damage may also lead to alteration of the protein structure and erroneous interpretation. The widely adapted solution to address this problem is cryo-cooling of the protein crystals, which increases the dose crystallographers can use to about 30 MGy at 100 K; the so-called Henderson limit where half the diffraction power of a typical crystal is lost.^216,217^ However, the maximal dose at which meaningful structural information can be retrieved remains a limiting factor, in particular, for challenging proteins where only small crystals are available (as for example membrane proteins^218^ or for proteins containing radiation sensitive metal clusters in their catalytic sites).

X-ray free electron lasers allow structural biologists to avoid the dose limit by providing extremely intense X-ray pulses of ∼10^12^ photons/pulse focused within a ∼1 *μ*m spot and a pulse length of 10–40 fs. Since the Henderson radiation damage limit is vastly exceeded within a single pulse, the sample rapidly explodes upon exposure. Yet, the pulse length in the femtosecond range is shorter than the appearance of any structural change within the protein, which allows to outrun the radiation damage during data collection.^219,220^ This “diffraction before destruction” principle allows the collection of data principally free of radiation damage and for the first time, it has become possible to measure static protein crystals at ambient temperatures without compromising the resolution and integrity of radiation sensitive catalytic sites.

An irony of the “diffraction before destruction” principle is that it hinders collecting multiple images of a single diffracting volume (crystal). This made it necessary to develop new ways of collecting data, commonly termed as serial femtosecond crystallography (SFX) because a large number of crystals (often thousands) are exposed in a serial fashion to the femtosecond XFEL pulses. Methods to deliver crystals include the fixed target approach, where crystals are scanned on chip-like devices,^109,221^ or the drop-on demand method relying on acoustic droplet ejection coupled with a conveyor belt drive.^222^ To date, the most successful and prominent method is based on a continuous flow of nano- to micrometer sized crystals injected with a jet device.^87,223^ The jet creates a continuous stream by pushing a crystal solution with high pressure through a thin nozzle and stabilizing it with a stream of gas. Initial versions of this method were relying on fast flowing liquid jets with high sample consumption,^97^ making the method suitable only for proteins that can be crystallized in large quantities. Sample consumption can be reduced by synchronizing the injection with the XFEL pulses^224^ or by slowing it using high viscosity injectors.^95^

A distinctive advantage of the latter method is that it can be slow enough to allow serial injection at the synchrotron, in a method dubbed serial millisecond crystallography (SMX).^102^ In this case millisecond exposures do not allow to outrun radiation damage as with the femtosecond XFEL pulses, but the dose is spread over thousands of small crystals keeping it at negligible levels even at room temperature. The key advantage of working at ambient temperatures is the possibility to study the dynamics of proteins in a time-resolved experiment. It is a long standing dream of structural biologists to go beyond the study of static protein structures at atomic resolution and to collect dynamical information, which was so far only accessible by indirect spectroscopic methods.^225^

**FIG. 5.**
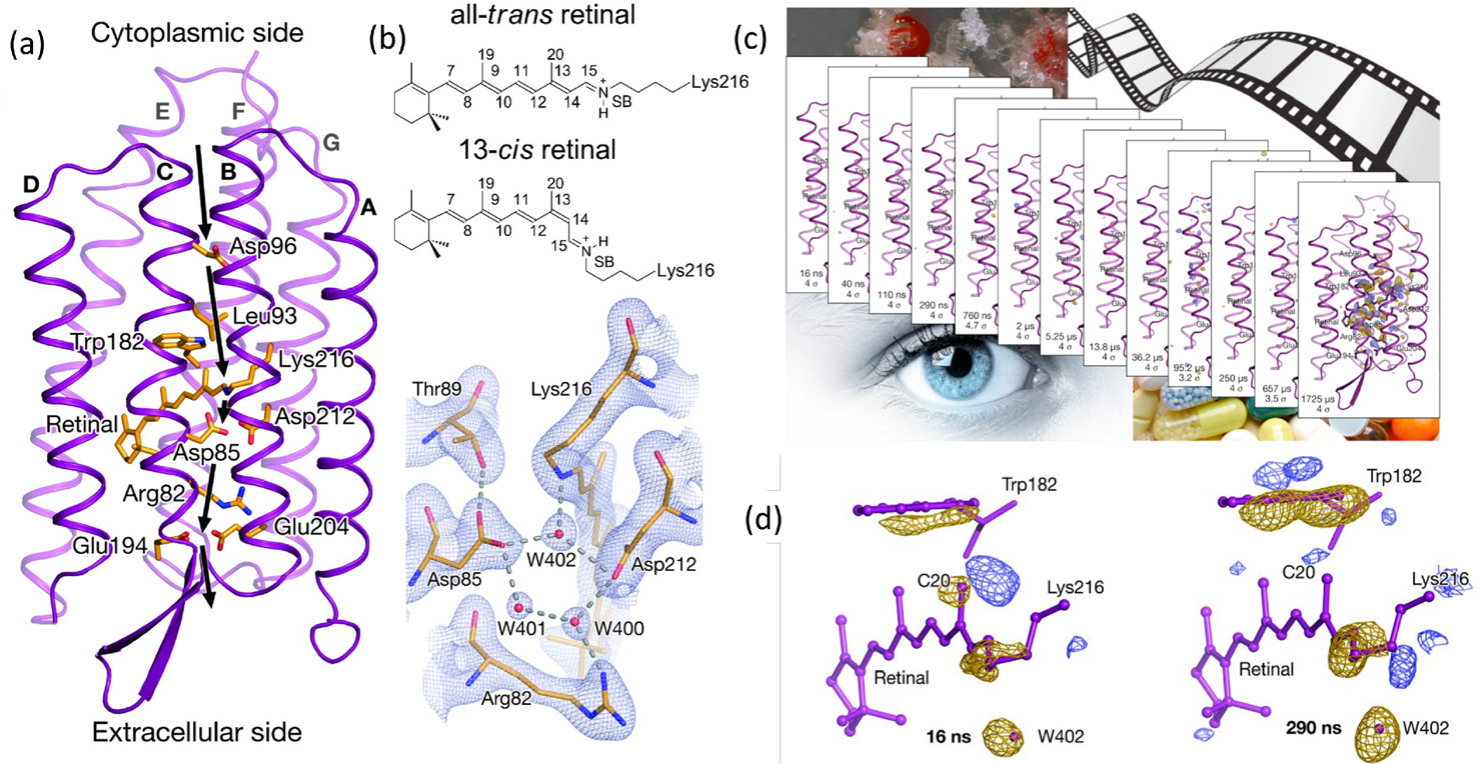
Serial crystallography at free electron lasers revealed the importance of water 402 in the early stages of bacteriorhodopsin (bR). (a) Structure of bR with a schematic outline of the Proton-exchange steps (arrows) achieving proton pumping by bR. (b) Schematic illustration of retinal covalently bound to Lys216 through a protonated Schiff base (SB) in an all-trans and a 13-cis configuration (upper panel) and 2mFobs – DFcalc electron density for the bR active site in its resting conformation. Electron density (gray) is contoured at 1.3 s (s is the root mean square electron density of the map). W400, W401, and W402 denote water molecules (lower panel). (c) Overview of all time points obtained with serial crystallography. (d) View of the |F_obs_|light – |F_obs_|dark difference Fourier electron density map near the retinal 16 and 290 ns upon activation. Blue indicates positive difference electron density, yellow a negative difference. The strong decrease in electron density at water 402 × 16 ns is clearly visible and further increased at 290 ns indicating a complete disordering of this water during the early activation phase. Reproduced with permission from Nango *et al.*, Science **354**(6319), 1552–1557 (2016). Copyright 2016 AAAS.

In a time-resolved serial femtosecond crystallographic experiment (TR-SFX), a given protein is activated at varying time points before being hit by the X-ray pulse of the XFEL allowing for the structural identification of reaction intermediates.^226^ Several examples of time-resolved serial crystallography have been published in the last few years covering a wide variety of time regimes ranging from a few hundred femtoseconds^88,227^ to several milliseconds.^100,228^ The “diffraction before destruction” principle makes it possible to work at ambient physiological temperatures, avoiding cryo-cooled crystals. The controlled variation of temperatures in a time-resolved experiment will furthermore allow a thorough and direct characterization of the energy landscapes of protein activation.^229^ Structural intermediates of proteins have previously been studied by freeze-trapping in crystals; the most extensively studied example likely being the light-driven proton pump bacteriorhodopsin (for a recent review, see Ref. 230). However, using this method no direct time information is obtained, while in addition the radiation damage and the overlapping structural intermediates make a conclusive interpretation of these data difficult. The recent use of TR-SFX on bacteriorhodopsin has provided a series of temporal snapshots that allow us to follow much more precisely how proton pumping is orchestrated in time and space.^100^ For example, it became clear why it takes several microseconds until a proton is pumped as this time is needed to fully reorganize the water clusters involved in the proton transfer pathway. In addition, it also clarified a dispute about the role of water 402 in the early stage of the activation process. It is now clear that at 16 ns water 402 is disordered, destabilizing the protonated Schiff base (SB) to Lys 216 (Fig. 5). It now seems reasonable to expect the application of this method in the near future to a series of similar retinal binding proteins including ion pumps, channels, and the visual photoreceptor rhodopsin.^231–233^

Another outcome of the power of XFELs to study the dynamics of radiation-sensitive biological systems is the photosystem II (PSII) protein complex, which is critical in plant photosynthesis. PSII catalyses the light-driven water splitting process, which maintains the Earth’s oxygenic atmosphere. In this process, the oxygen-evolving complex within PSII cycles through five states, S_0_ to S_4_, in four light-driven charge-separation events. Critical for the reaction is a Mn_4_CaO_5_ cluster in the catalytic site of the protein, which, like many catalytic metal clusters, is highly sensitive to radiation damage. High resolution and damage-free structures from XFELs have provided new insights into how the cluster is organised^234,235^ and, perhaps even more importantly, elucidated changes upon controlled flash illumination.^234,236–238^ All examples for TR-SFX mentioned earlier are natively light-activated proteins. Such a light trigger is necessary because currently the method depends on a pump laser pulse to initiate the reaction in a coherent way throughout the crystal. Even in the case of myoglobin, whose ultrafast dynamics have been extensively studied using both conventional Laue diffraction^239^ and TR-SFX at an XFEL,^88^ a laser pulse was used to trigger the release of carbon monoxide from the heme ligand, even though the protein is not natively driven by photochemical reactions like PSII, bR or the PYP studied in other recent TR-SFX experiments. The future of time-resolved pump-probe crystallography using XFELs, therefore, largely depends on whether triggers can be found to allow the study of not natively light-activated proteins. One promising way to achieve this goal is to chemically modify natural protein ligands so that they undergo a photochemical cis/trans isomerization similar to the natural ligands found in bacteriorhodopsin and the photoactive yellow protein. Such photoswitchable ligands are central to the field of photopharmacology, where they are employed as a non-invasive regulatory element for minimizing side effects of molecular drugs.^240^ In this way, a drug can be present in a state of low affinity when this is beneficial to reduce adverse effects. Later the drug can be switched to a state of high affinity when it has reached its site of action. Light is an ideal trigger for the switch, as it can be administered with high spatial and temporal precision but has otherwise very little effects on chemical and biochemical systems. The most common artificial photoswitches contain an azobenzene group, which allows cis-trans isomerization within a few picoseconds.^241^ The study of this ultrafast process and the effect the initial photochemical reaction has on the photopharmacological target protein will be a highly rewarding and medically relevant application for TR-SFX. Similar to the photoinduced release of CO from myoglobin,^88^ azobenzene photoswitches would, for example, allow to track the release of ligands from a wide range of proteins such as tubulin^242^ or G protein-coupled receptors.^243^ Combined with the computational molecular dynamics simulations,^244^ this will allow to study the dynamics of ligand recognition; the fundamental principle underlying the biological processes such as enzyme catalysis, molecule trafficking, and cellular signalling.

Another approach to overcome the current limitation to light-inducible reactions is to study ligand binding or enzymatic reactions by “mix and inject” serial crystallography.^245,246^ In this approach, a ligand solution is mixed with the crystals just before injection and data collection. This allows to study the binding of ligands and possibly even enzymatic reactions if these are slower compared to the diffusion time of the ligand in the crystal. Here, the possibility to obtain sufficient diffraction data for structural analysis from micron or even sub-micron crystals may turn out to be another decisive advantage of XFELs. This is because in such small crystals it seems much more feasible to initiate reactions in a concerted way as the reduced crystal size drastically decreases the diffusion time of the substrate into the crystal. In the case of membrane proteins, the use of 2D crystals for XFEL diffraction experiments^247–249^ may be highly advantageous for this purpose as a single layer of an ordered array of proteins would decrease diffusion times to the absolute minimum. This will grant easy access to the ligand binding sites and in addition allow measurements in a membrane like environment.

The calculations show that depending on the size of the crystal and the concentration and type of ligand under investigation, reactions ranging from several hundred milliseconds to seconds seem feasible with the “mix and inject” approach.^250,251^ Some even estimate that it would be possible to measure reaction times below 100 *μ*s for submicron-sized crystals,^229^ an initiation time which would cover a wide range of biological reaction. So far, however, the “mix and inject” systems are still under development as the time needed for mixing rather than diffusion within the crystal is still a limiting factor. In the two recently published examples for mix and inject serial crystallography, the fastest delay times were in the order of several seconds in case of binding of adenine to a RNA riboswitch^246^ and the antibiotic ceftriaxone to β–lactamase.^245^ A promising approach to further increase the time resolution is to decouple diffusion and mixing times from the actual reaction event by the use of photolabile (or photocaged) compounds.^252^ These are mixed with the crystals in an inactive precursor form, which can be activated by a laser pulse. This would increase the time-resolution into the low millisecond range^253^ and allow for the analysis of biological systems activated, for example, by ATP or the binding of specific ions. Faster reactions, however, will likely remain the domain of pump-probe approaches using pre-bound natural or artificial photoswitches.

In summary, the highlight of the initial phase of the time-resolved biological applications at XFELs has been dominated by the development of TR-SFX. This allowed measuring protein dynamics of directly light-driven reactions. The unique capability of XFEL pulses to outrun radiation damage and to obtain sufficient diffraction from micron to submicron-sized crystals (which can be more homogenously activated by light) in combination with the pump-probe method has allowed to probe structural snapshots from 100 fs to several milliseconds after light activation. This method is a unique opportunity to study the structural dynamics of light-activated proteins, including the large class of retinal binding proteins with their membrane ion pumps, channels, and G protein-coupled receptors.

Reactions that are not natively driven by light or can be activated using artificial photoswitches, can be approached by the mix and inject methodology. Yet for the foreseeable future, the time-resolution will likely remain limited to milliseconds for systems without a photoswitch as the initiating reaction has to be triggered in a concerted way throughout the crystal. The use of artificial photoswitches, photocaged compounds, or temperature jumps to initiate the reaction within the crystal is actively investigated throughout the community and may be a realistic option to dramatically widen the scope of time-resolved serial crystallography in the near future. Given that this field is only about 5 years old and extrapolating past successes into the future, we predict that the method will become a real game changer with respect to our understanding of protein dynamics. It should also be noted that it seems highly likely that the method will be adapted for use at conventional synchrotrons and at the next generation diffraction limited sources currently under planning and construction throughout the world.

## VI. APPLICATIONS TO NON-LINEAR X-RAY SCIENCE

**FIG. 6.**
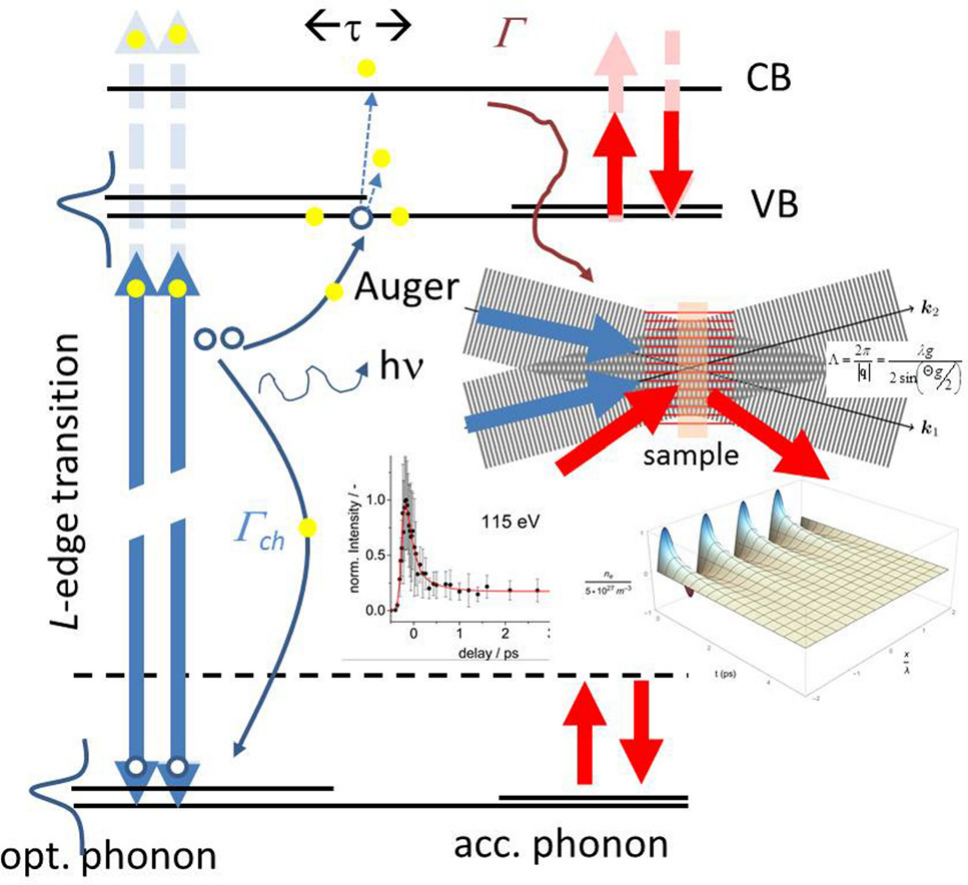
Schematic of an XUV-TG FWM excitation, using an optical probe and the XUV wavelength at the Si L-edge. Inlays depict a schematic of the grating formation by the two pulse interference and a typical XUV-TG signal from Si_3_N_4_.

With the advance of free electron lasers, the methodologies of nonlinear and quantum optics can be extended to the X-ray regime, employing the benefit of specific sequences of light-matter interaction with X-ray pulses. In general, this provides a high flexibility and selectivity in the experiments due to momentum and energy conservation of the interacting photons with the material. Most applied nonlinear X-ray processes, including parametric down-conversion,^254^ X-ray/optical SFG,^255^ stimulated emission/Raman,^256,257^ two-photon absorption (TPA)^258,259^ and emission,^259^ and two-photon ionization^260^ are rather static approaches, detecting the characteristic (e.g., spectrally resolved) photon or electron emissions at increased X-ray photon intensities. To date, the time-resolved non-linear X-ray methodologies that comprise temporal information are not so common and advanced experiments with ultrafast fs time resolution are accomplishable at FEL facilities only. As such, SwissFEL at Paul Scherrer Institute will provide an inimitable environment for moving in this direction in the near future. In the optical regime, nonlinear methods have been exploited extensively for molecular dynamics, control, and multidimensional correlation spectroscopy and for practical applications, ranging from advanced materials characterization and metrology to optical coherence tomography and biomedical imaging to coherent optics used in photonic switching and communications. Common X-ray spectroscopic methods provide a high locality and involve single-particle transitions between the core and valence electronic orbitals and the response to the transiently created core-hole state. Quantum coherence, which can be revealed and employed by non-linear spectroscopy, typically plays no role in linear X-ray techniques. With almost atomic resolution and specification, the locally induced energy and coherence can relax via intra- and intermolecular pathways and as such one may speak of relaxation and transport phenomena. Complex many-body quantum systems involve electron and nuclei motion and/or the evolution of quasi-particles such as phonons, excitons, magnons, or plasmons. Collective relaxation and decoherence gives rise to often complex dynamics. Unambiguous association of the fast (fs) time and short (nm) length scales of the electronic and collective structural rearrangements requires measurements addressing both regimes simultaneously. Nonlinear spectroscopy has the capability to measure quantum state correlations and thus provides information about coupling mechanisms, coherence, and the dynamics. Wave-vector dependent time- and energy-resolved X-ray four-wave mixing (FWM) allows, e.g., the preparation, manipulation, and readout of massive quantum superpositions. Due to the spatial and temporal dependence of the signals detected, X-FWM comprises additional information compared to standard pump-probe experiments. FEL based time-resolved XUV-four wave mixing (XUV-FWM) performed in a transient grating (TG) configuration has been recently demonstrated by Bencivenga *et al.*^261,262^ In this particular FWM-configuration, two spatially coherent crossed FEL pulses generate an interference pattern of the photon density on the sample (Fig. 6). The pattern periodicity is defined by the crossing angle and wavelengths of the X-ray pulses and thus can reach the nm range for hard X-rays. The X-ray photon absorption results in a periodic local modulation of the electron density and the depth profile is defined by the optical penetration into the sample material. The periodicity of the TG can be matched to system-relevant distances and allows for investigation on micro- (atom, molecule, lattice, and quantum mechanics), meso- (spin-, electron-systems, complex matter- and bio-systems, and many particle coherence and dephasing) and macroscopic length scales. Especially, the transition from the micro- to the mesoscale related to the electrical and magnetic properties of the material is of high interest in solid state physics. For example, in solid conducting or semiconducting materials, the arriving FEL photons produce free electrons and excite electrons locally from the atomic inner shells to the conduction-band. The inner-shell electron excitation is typically followed either by X-Ray fluorescence or Auger decay, resulting in the emission of another electron in the conduction band (CB) of the material. The electrons rapidly thermalize and free electrons can penetrate deeper into the material (≲200 fs). For d-band metals, e.g., the electron mean free path is in the order tens of nm. At longer distances, the electron transport would no longer be ballistic and the electron diffusion carries the energy further into the system, on a much slower time scale (∼1 ps). In semiconducting materials, the mean free path of energetic electrons is much shorter. In addition, highly energetic electrons in the CB can excite electrons from the VB through electron impact ionization. Thermalization between the VB and CB takes a longer time than carrier thermalization in the CB. However, the thermalized charge carriers are still out of equilibrium with the lattice phonons that are generated via electron-phonon interaction. At room temperature the coherence length of a phonon is in the order of nm and phonons are typically thought of as quasi-particles with velocity, density, and collision properties. All effects, occurring on different time and length scales are expected to be visualized by q-dependent time and energy resolved X-ray FWM.^263^ The direct observation of core excitations in hard X-ray FWM is more difficult because of the short core-hole lifetime limited by Auger processes and stringent requirements for phase control of X-Ray pulses, which is in the focus of the development of advanced FEL facilities.^264^ However, polarization control of FEL generated X-ray pulses is commonly available and thus X-FWM potentially can be applied for magneto-optical detection, allowing the investigation of spin dynamics, by using specific polarization configurations. In collective spin precessions, the so-called magnons can decay either by spin relaxation or spin diffusion, which in the case of a TG-FWM experiment denotes spin transport from one stripe to the other.^265^ We remind that a general aspect of non-linear spectroscopy is its selectivity due to multiphoton transition and symmetry rules, which incorporates the mixing of X-ray photons and the mixing of X-ray with optical photons (visible, UV, IR, and THz) and the centro-symmetrical and non-centro-symmetrical properties (bulk, surface) of the sample. Tuning frequencies on and off, the molecular resonances selects the number of excitation pathways contributing to the signal formation. This feature could be well explored in resonant X-ray emission spectroscopy (RIXS) in order to address the otherwise optically dark states. In this respect, nonlinear time-resolved X-Ray stimulated Raman (SXRS) is often considered as the time-domain extension of RIXS.^266^ The question, whether frequency- or time-resolved approaches are the method of choice, is not only a matter of optimizing the pulse parameters towards the smallest achievable energy bandwidth (often with cost intensive low efficiency spectrometers in the X-ray regime) or pulse duration. Also, the correlation between the signal spectrum and the temporal dependence can be utilized for an improved data analysis and experimental practicability. The development and the application of single shot time-resolved non-linear methods providing the same advantages as the scanning techniques are of high importance to the FEL science community because the time-resolved ultrafast X-ray measurement techniques typically suffer from long measurement times due to detailed scanning delays or from high intensities limiting the exposure to samples because of destruction. To overcome these drawbacks one may apply variants of hybrid (femto/pico) X-ray FWM techniques that, e.g., can be expressed in a way that allows for a direct ptychographic reconstruction of the system response given that several spectra have been measured from a temporally long and therefore spectrally narrow-band probe pulse.^267,268^ Besides the great potential and high flexibility of nonlinear X-ray spectroscopies, cross-sections for the corresponding X-ray multiphoton processes are in essence not available and up to now no clear general picture is drawn, even for the “simplest” nonlinear X-ray processes like the absorption of two X-ray photons. Thus, the investigation also on static approaches, greatly contribute to the understanding of time-dependent non-linear phenomena for extending these techniques into the tender and hard X-ray regime. By utilizing the high energy off-resonant X-ray emission spectroscopy, the measurement of non-linear two-photon absorption (TPA) cross sections been demonstrated in the hard X-ray regime.^259^ The flexible operation modes of SwissFEL, spanning an energy range between 0.25 and 12.4 keV will provide a unique opportunity to contribute to the development and understanding of non-linear X-ray spectroscopies and phenomena and their application in the hard, tender, and soft X-ray range.

## VII. OUTLOOK

We have seen in Secs. II–VI that early work using XFEL sources has already made significant contributions in the fields of chemistry, materials science, and structural biology. Moreover, the unprecedented high intensities of X-ray pulses from these new machines are not only opening a new field of non-linear optics, which promises to make significant further contributions to chemistry, biology, and materials science, but also to the areas of imaging and sensing. The new instrumentation and capabilities offered by the SwissFEL are aimed at further advancing the frontiers of ultrafast X-ray science in each of these areas. Ultimately, these methods hold the promise of unique insight into key problems of current and future relevance to a wide variety of disciplines.
